# Supplementary material for: HAL-X: Scalable hierarchical clustering for rapid and tunable single-cell analysis
Source: PLoS Comput Biol. 2022 Oct 3;18(10):e1010349. doi: 10.1371/journal.pcbi.1010349 (PMC9560626; doi:10.1371/journal.pcbi.1010349)
Supplement: S1 Methods — (PDF) [file pcbi.1010349.s001.pdf]

SUPPLEMENTARY MATERIALS.

HAL-X: scalable hierarchical clustering for rapid and tunable single-cell analysis

James Anibal, Alexandre G. Day, Erol Bahadiroglu, Liam O'Neil, Long Phan, Alec Peltekian, Amir Erez, Mariana Kaplan, Grégoire Altan-Bonnet, & Pankaj Mehta

Supplementary Methods.

CyTOF mass cytometric profiling of Lupus blood samples.

Acquisition of single–cell measurements.

Fresh Blood samples from 50 Lupus patients and 25 healthy volunteers were collected. Samples were immediately processed to separate low-density and high-density neutrophils (LDN and NDN) using Histopaque gradient. LDN and NDN fractions were then collected and washed in prewarmed complete RPMI immediately prior to cell-surface staining and acquisition. All reagents were from Fluidigm unless otherwise noted. Cells were first incubated with a 5-μM solution of cisplatin in PBS to mark dead cells. The cells were then washed and resuspended in Maxpar Cell Staining Buffer (MCSB) (Fluidigm). Human Fc receptor blocking solution (BioLegend) was added to each sample and incubated for 10 minutes at room temperature. All metal-labeled antibodies were purchased from Fluidigm. Biotin anti–human CD115 (BioLegend) was detected with Qdot-streptavidin conjugate (Thermo Fisher Scientific). A table of the panel of antibodies used for our mass cytometric measurements can be found in S1. Antibodies were diluted in MCSB at concentrations validated for minimal channel spillover, added to each sample, and incubated for 30 minutes at room temperature. Cells were washed twice with MCSB and then fixed for 15 minutes in a 1.6% solution of paraformaldehyde (MilliporeSigma) in PBS. Cell intercalation solution was prepared by adding Cell-ID Intercalator-Ir into Maxpar Fix and Perm Buffer (both from Fluidigm) to a final concentration of 125 nM. Cells were incubated with cell intercalation solution overnight at 4°C and then washed once with MCSB and Maxpar Water twice. Cells were finally resuspended in a water solution containing EQ Four Element Calibration Beads (Fluidigm) immediately prior to CyTOF data acquisition at a cell concentration of 10^6cells/ml (¡300 events per second) and filtered into cell trainer cap tubes. Data were acquired on a Helios Mass Cytometer (Fluidigm) as previously described [1].

Data preprocessing.

Raw mass cytometric data were normalized with the Normalizer algorithm as recommended by the software developers. Data were first gated as live singlet cells using (EQ4–cisplatin) and ($CD45^+$DNA(2n)) manual gates (Supplemental Figure S2) and exported as Python dataframes. These dataframes were then processed with a custom–programmed pipeline in Python, based on an implementation of HAL-X.

Algorithm 1 HAL-x

Input: data $x_i \in \mathbb{R}^D$, desired out-of-sample accuracy c_v
if D large **then**
  Learn latent space (encoder, weights W): $z_i = f_W(x_i)$
  Perform dimensional reduction: $y_i = g(z_i)$
else
  $z_i \leftarrow x_i$
end if
Estimate density map: $\rho(z_i) \in \mathbb{R}$
Determine local modes of ρ**:** $\boldsymbol{\mu} \equiv \{z_k\}$.
Assign to each z_i **a unique mode** ($k = 1, \cdots, K$)
Construct density graph: $\mathcal{G} \equiv \{\mathcal{E}_{ij}, \mathcal{V}_i\}$**,** $i, j \in [1, K]$
for $i, j \in [1, K]$ **do**
  if μ_i is *density connected* to μ_j **then**
    $\mathcal{E}_{ij} = 1$
  end if
  $\mathcal{E}_{ij} = 0$ otherwise
end for
Coarse-grain density graph:
for $\mathcal{E}_{ij} \neq 0$ in $\mathcal{G}$ **do**
  compute out-of-sample score S_{ij} for modes i and j.
end for
while $\min S_{ij} < c_v$ **do**
  merge clusters with worst S_{ij}
  recompute graph G
  compute score S_{ij} for new edges
end while
Return Validated cluster assignments and set of classifiers.

Complexity for generating multiple clusterings

The scalability of HAL-x is facilitated by the supervised classifiers used to merge the clusters. We simply use the trained models to predict the cluster labels on the raw data. For multiple sets of clusters, we use classifiers at different levels of the hierarchy. The worst-case time complexity for prediction via a trained random forest is *O($v \times t$)* where v is the number of variables (features) and t is the number of trees in the forest. The worst-case time complexity for prediction via a trained SVM is *O($v \times fs$)* where v is the number of variables and s is the number of support vectors. With this sub-exponential runtime, HAL-x can easily predict labels for datasets with over 25 million cells, as we show in this report.

HAL-x algorithm

In Algorithm 1, we present the pseudocode representation of HAL-x model generation (end-to-end).

Supervised classification of single cells

First, we used random sampling to balance the data contained in each cluster, ensuring an equal amount of cells from Lupus patients and from healthy individuals. For each cluster, we used a random forest classifier (with bootstrapping) to predict the clinical status of the individual cells (Lupus or healthy).

Low dimensions benchmark datasets

To demonstrate the relationship between goodness of clustering and out-of-sample accuracy, we have generated 220 synthetic clusterings across 6 benchmark datasets shown in Fig. S4. Fig. S4 shows the ground truth clustering labels. We used K-means, DBSCAN, MeanShift and Spectral clustering from scikit-learn to cluster the benchmark datasets. We further used various hyperparameters to generate a variety of clustering labels to provide a wide range of situations to demonstrate the accuracy vs. good of clustering thesis.

Weighted F1-score

The F1-score is a metric for determining goodness of clustering. Given two sets of clustering labels, $\{Y_i\}_{i=1}^{N}$,$\{Z_i\}_{i=1}^{M}$ we can use the Hungarian algorithm `https://en.wikipedia.org/wiki/Hungarian_algorithm` in order to find the optimal pairing between the Y_i and Z_i clustering labels. The cost function used for the Hungarian algorithm is $1 - F$, where F is the F-matrix of dimension $N \times M$. Each entry (Y_i, Z_j) of the F-matrix is the corresponding F-score (harmonic mean of precision and recall). If $N \neq M$, as is generally the case, there is a many-to-one or one-to-many relationship. Once the mapping is established via the Hungarian algorithm, we can simply compute the final weighted F1-score by summing over all entries of the F-matrix that are returned by the Hungarian algorithm (i.e. the best map) and weight each F-score by relative size of the cluster considered. For more discussion around this we refer the reader to [2].

Robustness to embedding

In Fig. S5 we ran HAL-x clustering for the synthetic data using two different embeddings: t-SNE and UMAP. UMAP [3] has recently become a popular alternative to t-SNE due to it's computational speed and similar embedding accuracy. Fig. S5 shows the resulting clustering from HAL-x using a cv score of 0.8 when using t-SNE (a) vs. using UMAP (b). The clustering performance is identical since the underlying embeddings are structurally similar. We note however that UMAP is significantly faster than t-SNE. While using UMAP does not alter the conclusions provided in the main text, we have added the option to use UMAP for the embedding in the core HAL-x code.

Optional Hal-x functionality

In Fig. S3 we showcase a small optional dashboard functionality of HAL-x that allows the user to explore quickly the results of the hierarchical clustering. The dashboard allows the user to visualize the hierarchical relationship of the clusters w.r.t. the cv score.

References

1. Ravell JC, Matsuda-Lennikov M, Chauvin SD, Zou J, Biancalana M, Deeb SJ, et al. Defective glycosylation and multisystem abnormalities characterize the primary immunodeficiency XMEN disease. The Journal of Clinical Investigation. 2020;130(1):507–522. doi:10.1172/JCI131116.

2. Weber LM, Robinson MD. Comparison of clustering methods for high-dimensional single-cell flow and mass cytometry data. Cytometry Part A. 2016;89(12):1084–1096. doi:https://doi.org/10.1002/cyto.a.23030.

3. McInnes L, Healy J, Saul N, Grossberger L. UMAP: Uniform Manifold Approximation and Projection. The Journal of Open Source Software. 2018;3(29):861.
