# Supplementary material for: HAL-X: Scalable hierarchical clustering for rapid and tunable single-cell analysis
Source: PLoS Comput Biol. 2022 Oct 3;18(10):e1010349. doi: 10.1371/journal.pcbi.1010349 (PMC9560626; doi:10.1371/journal.pcbi.1010349)
Supplement: S1 Fig — (PDF) [file pcbi.1010349.s002.pdf]

Tag	Target	Clone
141Pr	CCR6	G034E3
158Gd	CD10	HI10a
116Cd	CD115[biot] + SA(Qdot)	
162Dy	CD11c	Bu15
151Eu	CD14	M5E2
144Nd	CD15	W6D3
170Er	CD152/CTLA4	14D3
209Bi	CD16	3G8
164Dy	CD161	HP-3G10
142Nd	CD181(CXCR1)	8F1/CXCR1
147Sm	CD182(CXCR2)	5E8 (CXCR2)
173Yb	CD184 (CXCR4)	12G5
159Tb	CD197(CCR7)	G043H7
166Er	CD24	ML5
155Gd	CD27	L128
168Er	CD278 [ICOS]	C398.4A
174Yb	CD279(PD-1)	EH12-2H7
160Gd	CD28	CD28.2
154Sm	CD3	UCHT1
145Nd	CD31 (PE-CAM1)	WM59
169Tm	CD32	FUN-2
163Dy	CD33	WM53
167Er	CD38	HIT2
089Y	CD45	HI30
165Ho	CD45RO	UCHL1
176Yb	CD56	NCAM16.2
153Eu	CD62L (L-Selectin)	DREG-56
150Nd	CD63	H5C6
149Sm	CD66a/c/e	ASL-32
152Sm	CD66b	80H3
156Gd	CD86	IT2.2
146Nd	CD8a	RPA-T8
195Pt	Cisplatin (Live Dead)	-
191Ir	DNA Intercalator 1	-
193Ir	DNA Intercalator 2	-
143Nd	HLA-Dr	L243
148Nd	IgA	Polyclonal
172Yb	IgM	RMM-1
