# Supplementary figures and images for: HAL-X: Scalable hierarchical clustering for rapid and tunable single-cell analysis
Source: PLoS Comput Biol. 2022 Oct 3;18(10):e1010349. doi: 10.1371/journal.pcbi.1010349 (PMC9560626; doi:10.1371/journal.pcbi.1010349)

**T**

Lu175Di :: 175Lu\_EQ4\_Be...

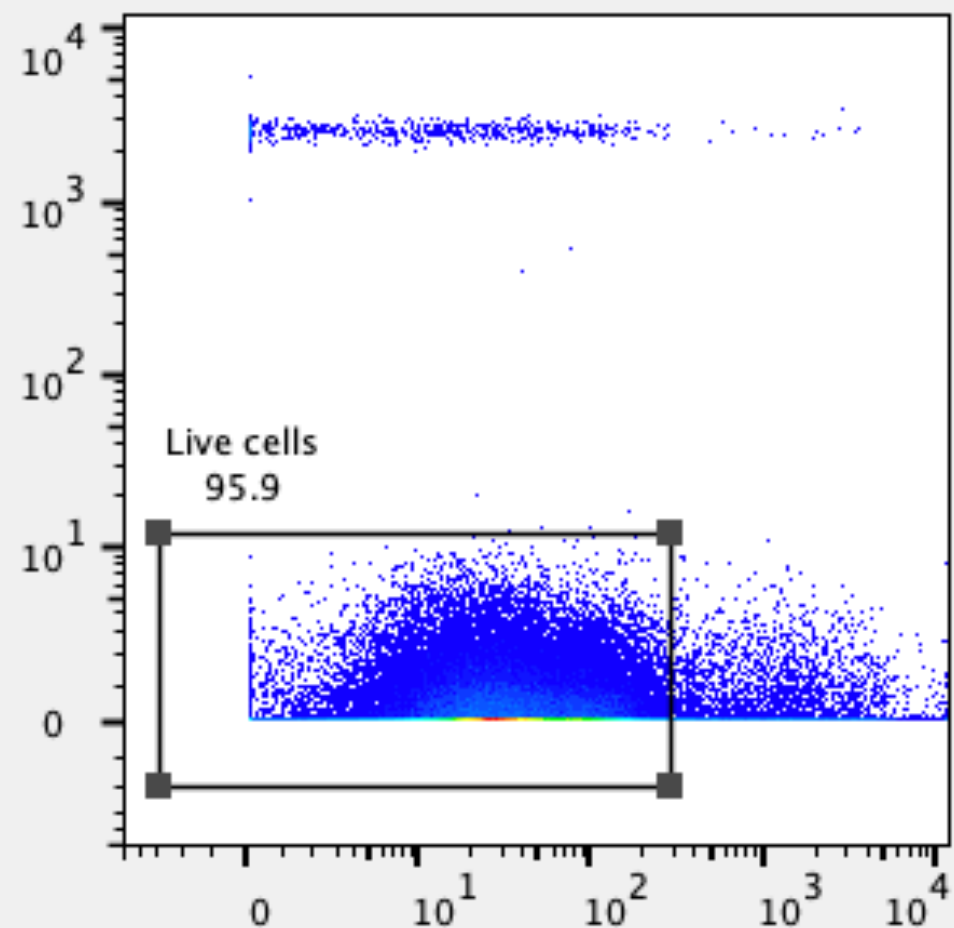

Pt195Di :: 195Pt\_LiveDead\_... ▼

**T****T**

Ir193Di :: 193Ir\_DNA

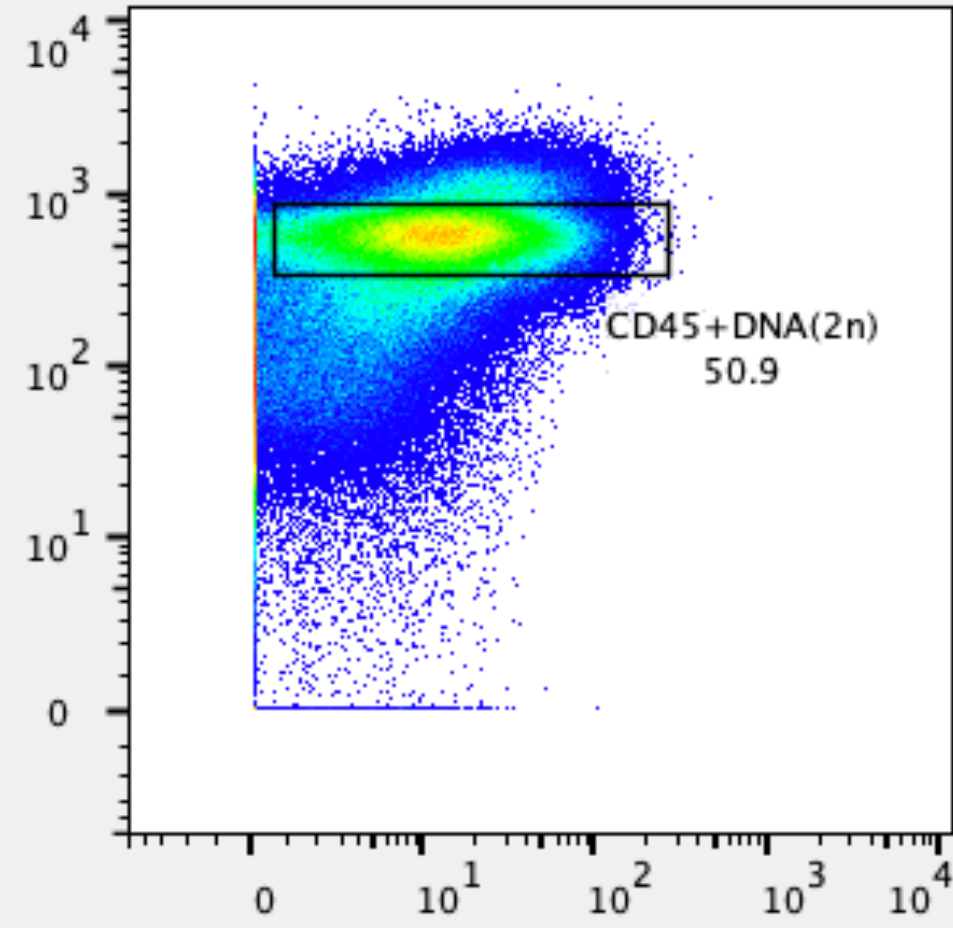

48, 1.72)

Y89Di :: 89Y\_CD45 ▼

**T**

Supplement: S2 Fig — Left: gating for live cells (excluding Cisplatin-positive dead cells and EQ4 calibration beads. Right: gating for CD45+DNA(2n) singlet leukocytes. (PDF) [file pcbi.1010349.s003.pdf]

Hierarchical Structure

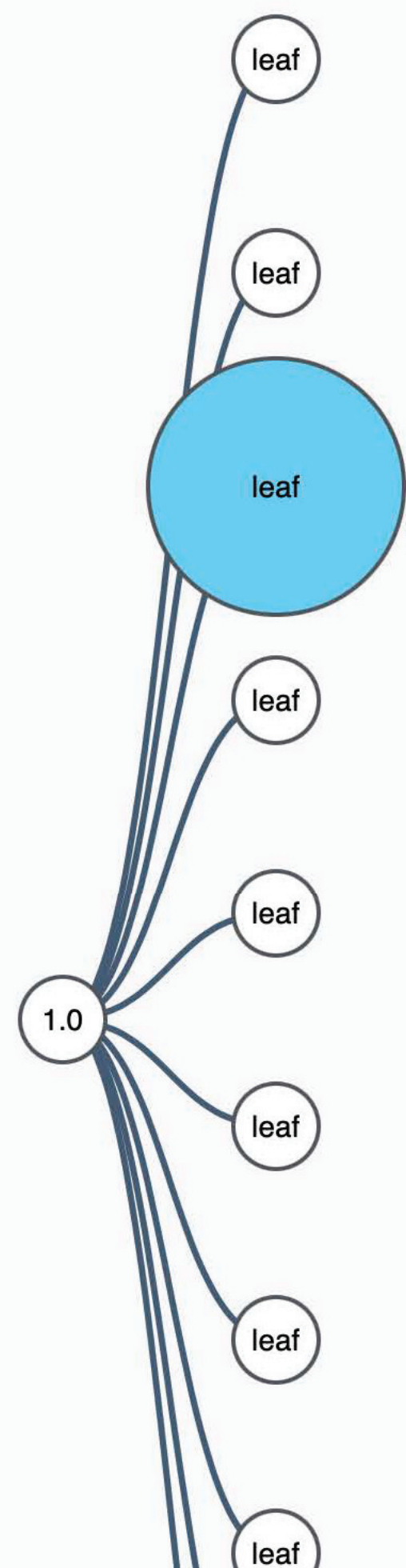

t-SNE ▾

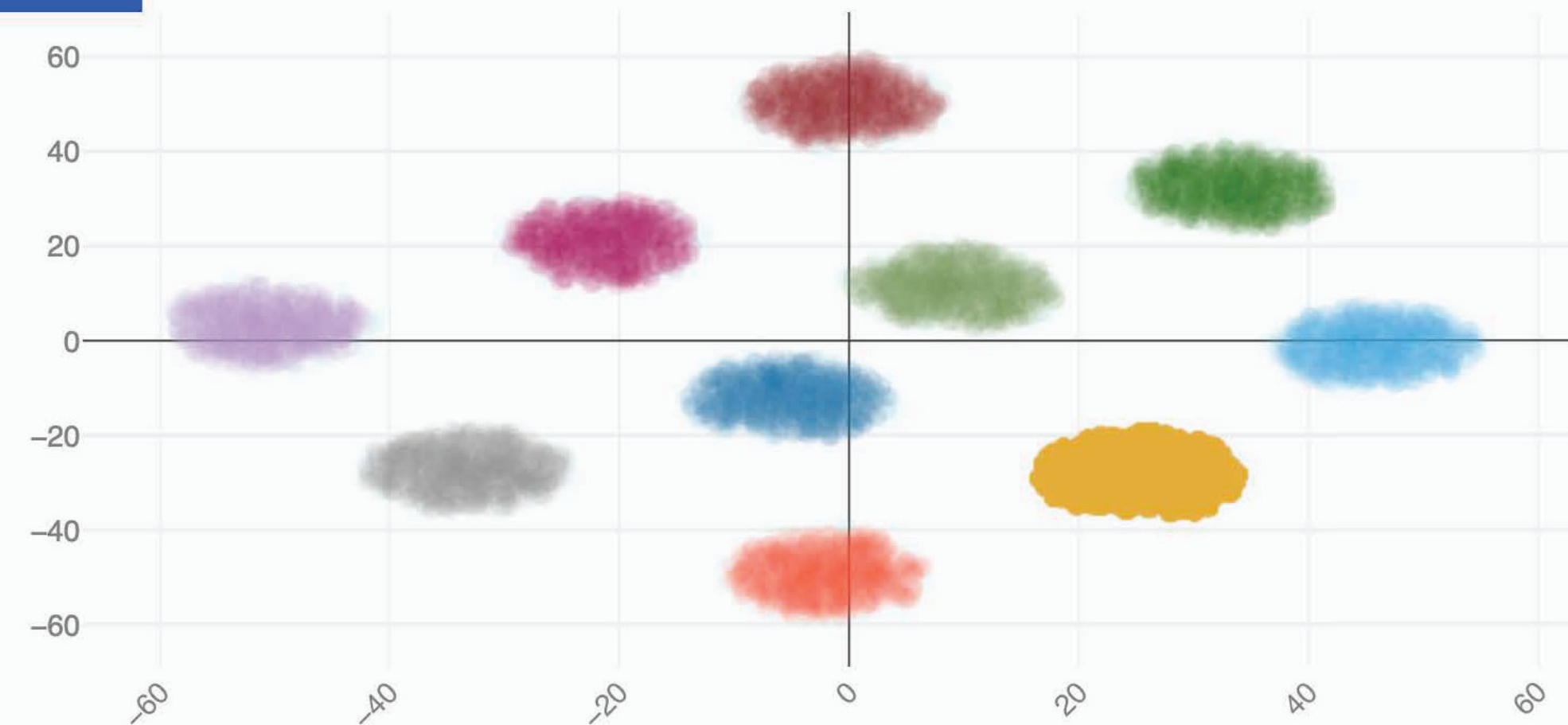

Median expression ▾

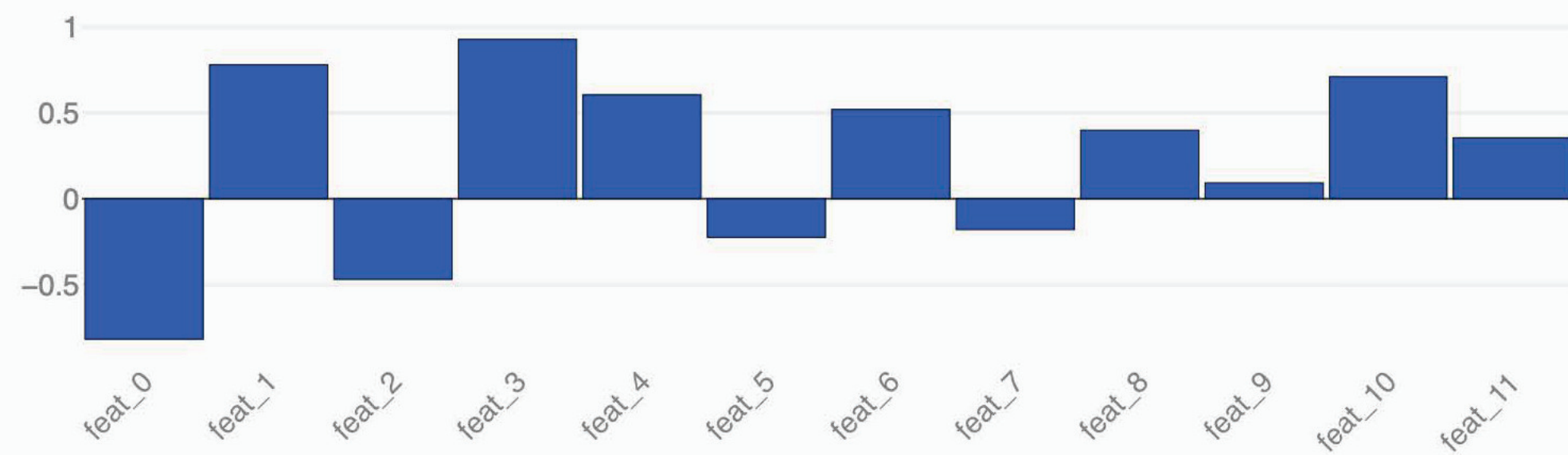

Supplement: S3 Fig — The dashboard has 3 panels. (left-side) The hierarchical relationship between the clusters. (top right side) The embedding map with the clustering labels. (bottom right) When choosing a cluster, this shows the profile of that clusters in terms of the original features. (PDF) [file pcbi.1010349.s004.pdf]

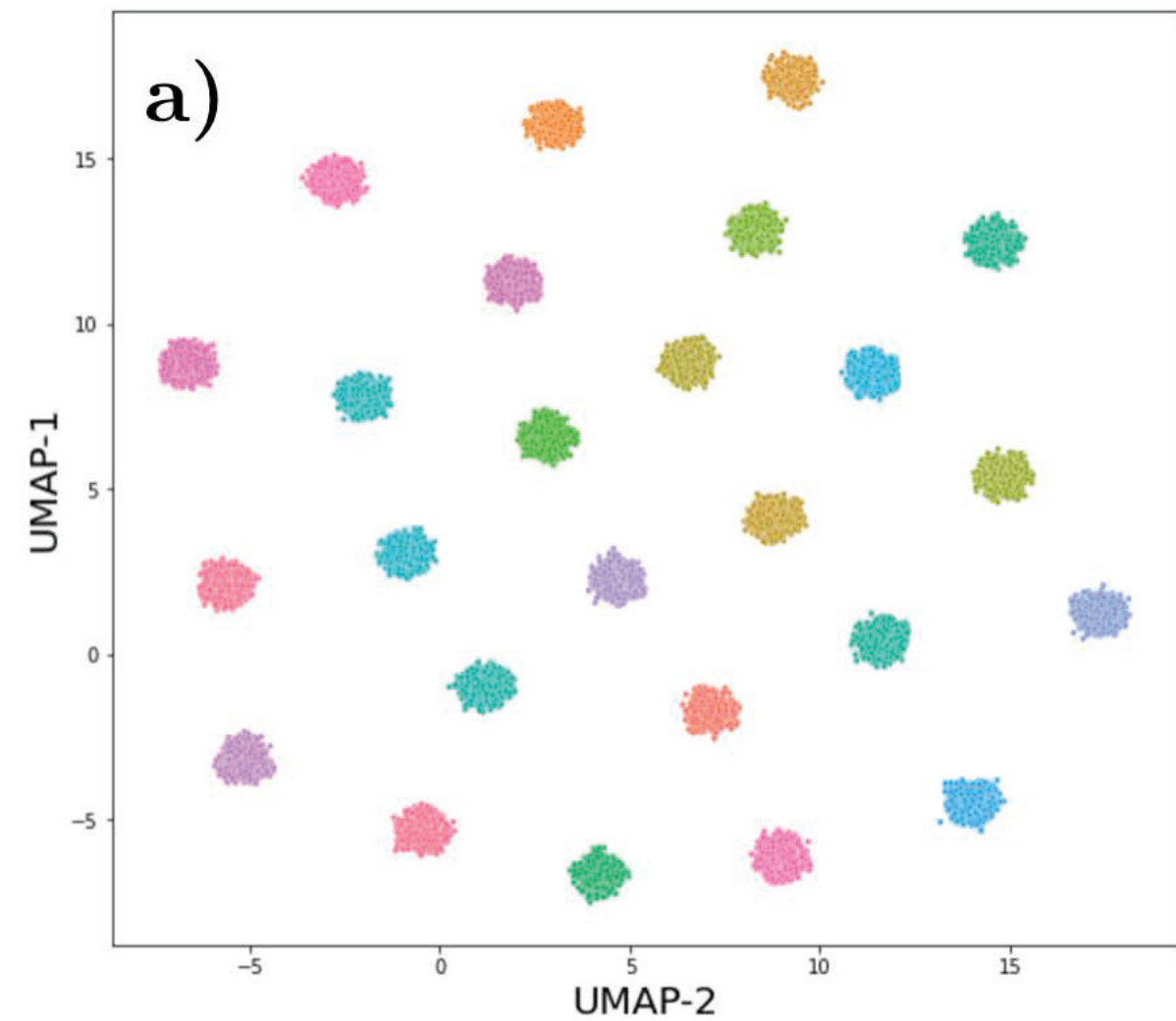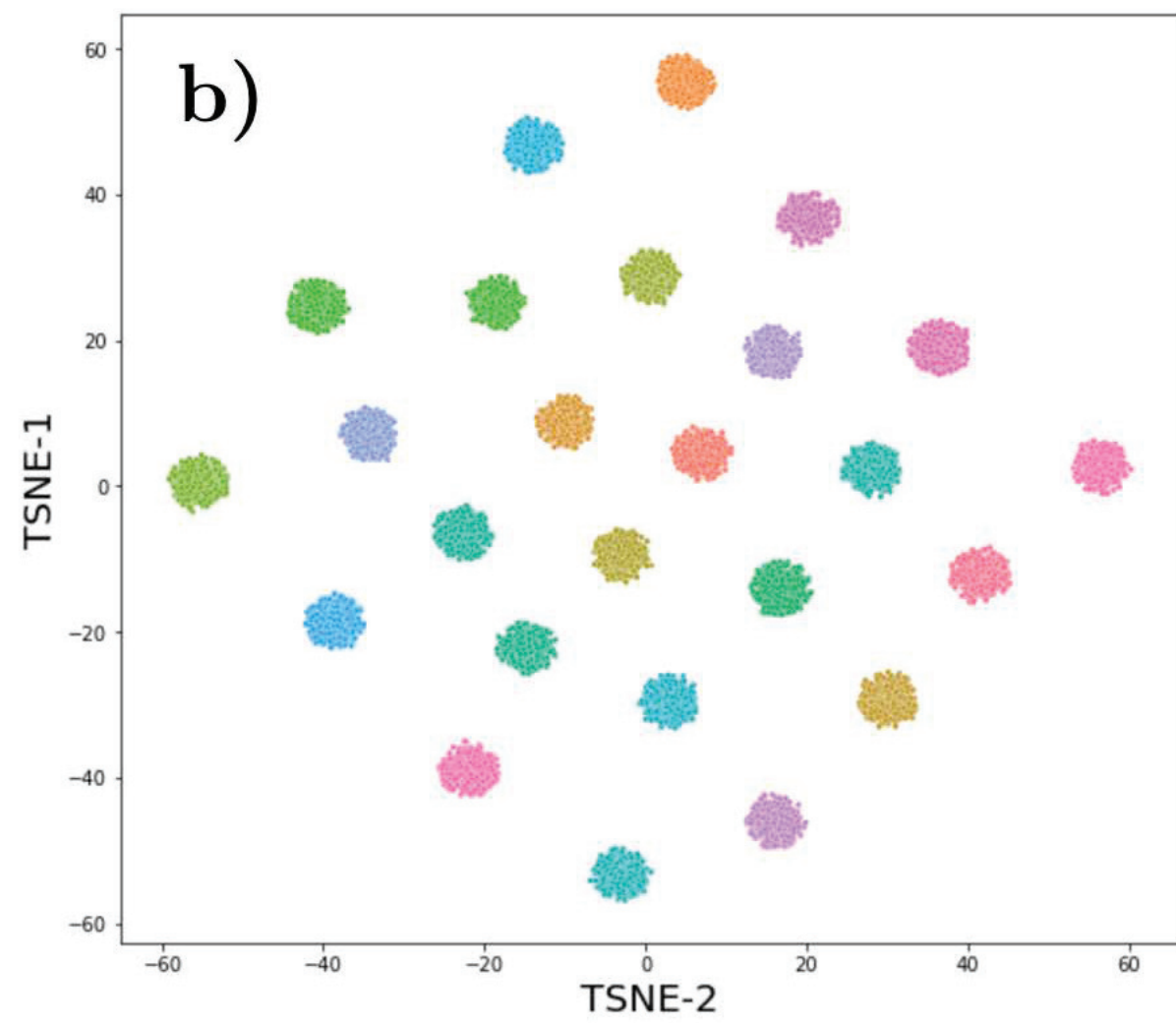

Supplement: S5 Fig — The results are very similar although we found that the UMAP approach ran approximately 50% faster in this instance. (PDF) [file pcbi.1010349.s006.pdf]
