# Supplementary material for: HAL-X: Scalable hierarchical clustering for rapid and tunable single-cell analysis
Source: PLoS Comput Biol. 2022 Oct 3;18(10):e1010349. doi: 10.1371/journal.pcbi.1010349 (PMC9560626; doi:10.1371/journal.pcbi.1010349)
Supplement: S4 Fig — The red/blue/green coloring represents the ground-truth clustering labels when generating the datasets. The datasets are taken directly from scikit-learn clustering benchmark page. See https://scikit-learn.org/stable/modules/clustering.html for the exact definitions of the datasets. (PDF) [file pcbi.1010349.s005.pdf]

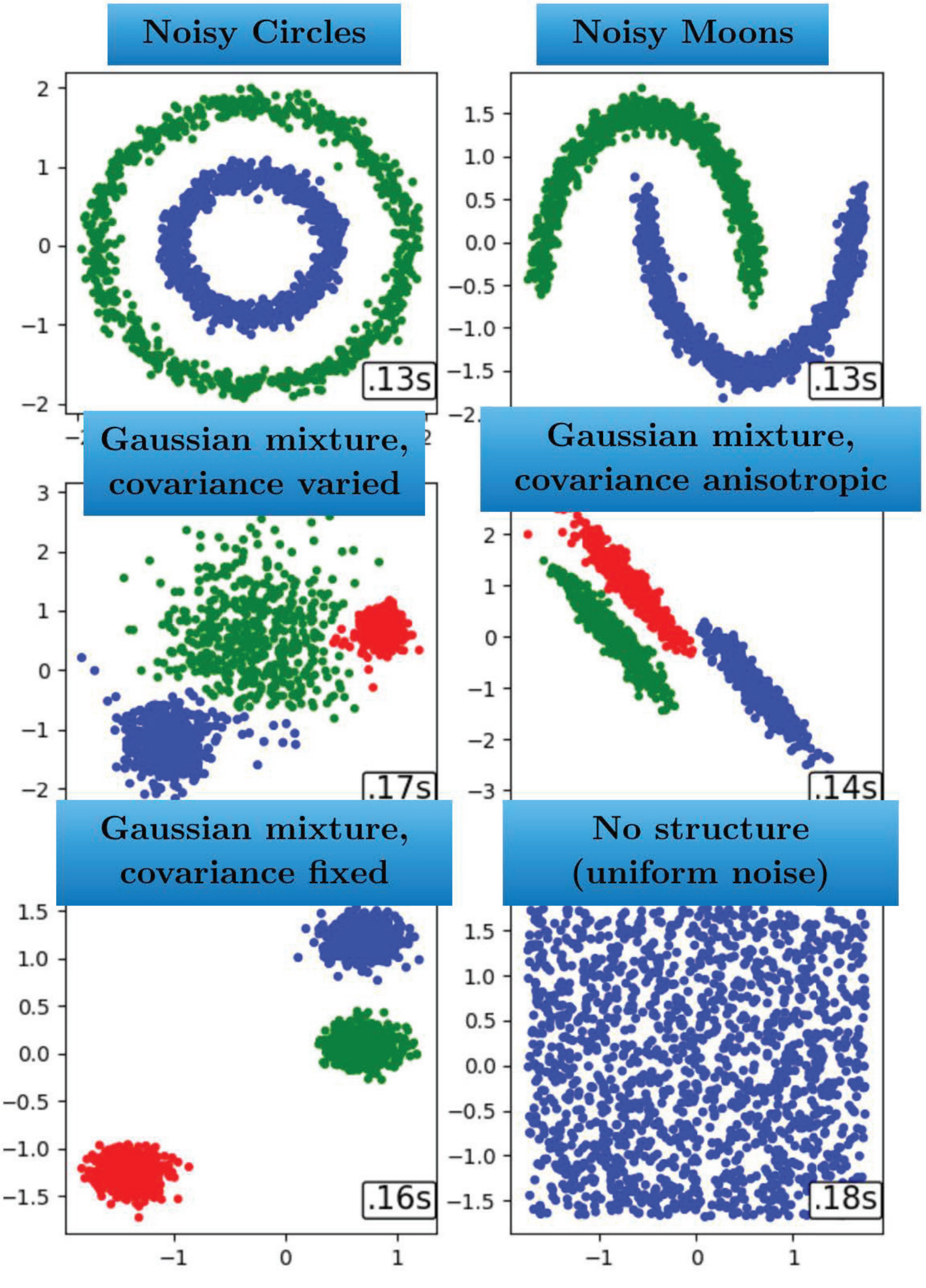

Noisy Circles
Noisy Moons
Gaussian mixture, covariance varied
Gaussian mixture, covariance anisotropic
Gaussian mixture, covariance fixed
No structure (uniform noise)
.13s
.13s
.17s
.14s
.16s
.18s
