# Supplementary material for: HAL-X: Scalable hierarchical clustering for rapid and tunable single-cell analysis
Source: PLoS Comput Biol. 2022 Oct 3;18(10):e1010349. doi: 10.1371/journal.pcbi.1010349 (PMC9560626; doi:10.1371/journal.pcbi.1010349)
Supplement: Attachment — Submitted filename: 20220307 Reviews @ PlosCompBio.docx.pdf [file pcbi.1010349.s007.pdf]

Dear Dr. Faeder & Marz,

Thank you for giving us the opportunity to rebusmit our manuscript (PCOMPBIOL-D-21-01438) entitled:

“HAL-X: scalable hierarchical clustering for rapid and tunablesingle-cell analysis”/

We thank the reviewers for their many excellent comments and suggestions. We have significantly revised the manuscript, including testing our algorithm on additional low-dimensional embeddings such as UMAP and performing additional tests using synthetic datasets. We feel that the manuscript is significantly improved and addresses the major concerns and questions of the reviewers. Please find below our detailed responses (in black font) to the referees’s comments (reproduced in italic/blue font).

We are looking forward to hearing your decision regarding this resubmission.

Sincerely,

Grégoire Altan-Bonnet & Pankaj Mehta

Reviewer's Responses to Questions

Comments to the Authors:
Please note here if the review is uploaded as an attachment.

Reviewer #1: The manuscript by Anibal, Day, et al. introduces a new data clustering method (HAL-x) that is well-suited for studying large, high-dimensional data sets like those generated by mass cytometry. Data clustering is an important and timely topic given the emergence of large data sets in biology that can contain tens of millions of data points in a feature space with 40 or more dimensions. There have been a number of data-clustering efforts in this area, which the authors review in their paper. The authors overcome shortcomings of existing works by creating a workflow involving nonlinear dimensionality reduction, density clustering, and supervised linkage methods to create a cluster hierarchy on raw data. This work advances the field through the computational efficiency (scalability) of the method and the ability to readily generate multiple clusterings at varied depths.

Major concerns

1. In the “Related Work” section, the authors describe a shortcoming when using nonlinear embeddings like t-SNE and then performing clustering in the low-dimensional space: Namely, that the low-dimensional representations can compress the data and lead to spurious clusters. The first step of the HAL-x method uses t-SNE on a down-sampled portion of the data. It would be useful for the authors to explore how the initial embedding in low-dimensional space impacts the subsequent clustering by their algorithm.

We primarily used FFT accelerated t-SNE because it provided speed of execution and reasonably good embeddings. Our agglomerative method is robust to changes in the low-dimensional embedding technique as

long as regions of high density in the generated embedding represent true clusters in the high dimensional space. In order to study the effect of different embeddings, we modified our source code to allow for the use of UMAP [McInnes et al, The Journal of Open Source Software, 2018]. We added the option embed_method='tsne' or embed_method='umap' to our HAL-X pipeline. We reran our clustering code for the synthetic dataset with the UMAP embedding and obtained F-scores similar to the ones obtained via t-SNE (see supplemental materials - Figure S5). We also note that we found our clustering method ran 2-times faster when using UMAP instead of tSNE. The robustness of results to the choice of UMAP or tSNE suggests that our clustering algorithm is thus not overly sensitive to the choice of low-dimensional embedding. The underlying reason for this is that the algorithm starts by ``over clustering'' and then merging clusters using out-of-sample classification scores in the *original high-dimensional* space. We are grateful to the referee for making this comment and making us explicitly test this assumption.

2. The authors discuss (page 7) the possibility of preprocessing the input using an encoder "if necessary." How can one decide if this step is necessary?

Preprocessing data is always a difficult but important consideration. We know of no principled way of answering this question. However, we have extensively tested this empirically on many biological and non-biological datasets. We have found that when dealing with CyTOF data, in general, there is no need to use an encoder. However, when applying the clustering algorithm to image datasets, using an encoder before performing a low-dimensional embedding does significantly improve the performance of our clustering algorithm. Since the paper is almost exclusively concerned with biological applications with single-cell measurements (e.g. CyTOF),. we removed any mention of the encoder in the text and in Figure 3.

3. Regarding Figure 1, I was not convinced that "clusterings with high F1-scores also tend to have good out-of-sample accuracy confirming that out-of-sample accuracy is a reasonable proxy for good clustering." Many clusterings with intermediate and even low weighted F-scores also have good out-of-sample accuracy. I encourage the authors to explain/explore this in more detail. I am not sure that I follow the logic/conclusion, or perhaps I am misunderstanding aspects of the figure.

We added clarifying figures to discuss the relationship between F1-score and out-of-sample accuracy. To do so, we computed the out-of-sample prediction accuracy of random forest classifiers (figure 1 and figure 2) on multiple benchmark datasets taken from the scikit-learn library of Python. We found a clear monotonic relationship between F1-score and the out-of-sample accuracy across multiple benchmark datasets and clustering methods as shown in figure 2.

4. The text in this section ("Out-of-sample error…") refers to supplemental materials that I cannot find.

We thank the referee for remarking on this. We have added all the linked information (scikit-learn datasets, github repository/source code used) in the supplemental information section. We have also made the full HAL-x Github repository public (https://github.com/alexandreday/HAL).

5. For the synthetic data set: How were the points distributed? How well did the algorithms perform on the data (i.e., how well did they cluster, assuming structured data was provided)? It seems like there is additionally opportunity here to assess the performance of the method.

For the synthetic dataset, we used the scikit-learn "make_blobs" function to generate 25 isotropic Gaussian blobs, each with 30 features, akin to the structure of single-cell data. These data are a very straightforward clustering task,

resulting in near-perfect accuracy values. Our use of the “make_blobs” synthetic dataset was dedicated primarily to assess runtime, not performance, compared to other clustering algorithms (HDBSCAN, Phenograph & Parc).

6. I recognize that this is a methods-oriented paper. However, I think the paper would be strengthened by expanding the discussion of results for the Lupus dataset. For example, “if we had fixed the cv-score at 0.8, we would have missed several biomarkers and lost valuable information about the cellular landscape.” The authors don’t have to dramatically expand the scope of their paper, but this really leaves me hanging as a reader. I would like to know more about the biomarkers and/or what types of clusters are merged as cv-score increases.

We expanded our discussion of our findings for the Lupus dataset. Indeed, we had limited this discussion to the minimum but we understand that the readership of PLoS Computational Biology necessitates more discussion on the biological implication .

We now describe how clustering of the leukocyte subpopulations at different depths can lead to a biomarker that distinguishes blood samples from Lupus patients from blood samples from healthy donors (HD). This signature relates to the difference between lupus and HD within a cell type. For example, in our specific case, we identified a cluster of neutrophils ($CD15^{+}CD16^{+}CD32^{+}CD66ace^{+}CD66b^{+}$) that exhibit different expression patterns between lupus and HD. Significant biomarkers include CD16, CD10, CD24, and IgA. Note that the clustering needs to be done at appropriate depths in order to capture clusters which contain statistically-significant differences between patients and HD: using too high a cross-validation score limits the number of clusters and fails to capture the clinical difference between Lupus patients and HD; using too low a cross-validation score increases the number of clusters but their robustness becomes limited and their statistical significance stunted (this also illustrates the importance of proper correction for false positive observations).

In regard to the biological significance of our findings, neutrophils are very sensitive to altered inflammatory milieu (in particular, because their production and replenishment occurs over less than 2 days). Lupus being an autoimmune inflammatory disease, it is not surprising to find neutrophil alteration as a good biomarker. Proper comparison with other autoimmune disorders and testing in animal models will be necessary to derive more empirical understanding of the observation derived from our algorithm here.

We generated a more detailed Figure describing our Lupus results and expanded on the statistical & biological relevance of our findings in the main text.

Minor comments

- Some terms are not defined in Eqns 1 and 2.

We added an explicit description for each term and updated the manuscript accordingly

- Page 9: What does “consider the full set of pure clusters” mean?

“Pure cluster” is a term often used to describe the initial clusters in agglomerative clustering methods. The purity of a cluster is the extent to which a cluster contains a single class [**see, Introduction to information retrieval, Schutze et al. Cambridge University Press Cambridge, 2008**]. To make this, more clear, at the beginning of section ‘Identification of Pure Clusters’ we provide our definition of ‘pure’ clusters:

“HAL-x identifies an initial set of ``pure'', high-density clusters that form the starting point for agglomerative clustering.”

Later in the text we have also made this clear:

“After the initial clustering (i.e. after the identification of the ``pure'' clusters), HAL-x performs agglomerative clustering by linking clusters that are hard to distinguish.”

- Is FlowCAP I the same as the Levine benchmark datasets?

The datasets are different. We used the “Normal Donors” dataset from FlowCAP I (it consists of a 10-parameter flow cytometry datasets). The Levine benchmark is a CyTOF dataset analyzing blood samples from AML patients using a 31-antibody panel (Levine et al., Cell’2015).

A more complete description of the dataset from FlowCap I can be found here:

http://www.flowcap.flowsite.org/

We also edited the manuscript to add further descriptions in order to clarify.

- Based on the wording, it was hard to tell if the lupus data was generated for this paper or was a previously published data set.

Our Lupus dataset has not been published before. It represents a typical challenge for clustering on large(r)-scale datasets (> 20 Millions cells) and highlights the strength and versatility of HAL-X. We are now making the dataset available on the Zenodo repository (10.5281/zenodo.6332934)

Typos
“tend to have out good of sample” (page 5).

“that are only slightly differences” (page 8) .
“with 10 millions data” (page 11)
“constitute a good biomarkers” (page 12)

Thank you for pointing these out: we corrected these typographic errors.

Reviewer #2: In this paper, Anibal et al. describe a novel method for data clustering, especially high-dimensional data such as that produced by CyTOF and similar experimental methods. Their method works by reducing the dimensionality of the data, developing a base set of clusters on this space, and then

refining them. One of the key principles in this work is that a good clustering algorithm should produce clusters that generalize well to new data. The authors test their method against other existing approaches and find that their approach has advantages in both speed and accuracy.

This paper makes a valuable contribution to the literature on practical clustering methods. However, there are a number of points where the paper could be clarified or where important concepts are underexplored. In general, some details of the methods and simulations are unclear or have not been given, and the examples on real data could be explored further. My detailed comments are below:

1. I ran into several problems attempting to run the authors' software, at least if this is run outside of Anaconda. In order to use the software, users must have the FFTW package installed because it is a requirement for fitsne. However, this is not currently mentioned in the installation notes, and a naïve installation through pip will fail without FFTW. The authors should note the dependency on FFTW and point to where it can be installled. The posted example also fails with the current version of sklearn due to changing conventions in the make_blobs function. I recommend that the authors change that line of the example to: "X,y = make_blobs(n_samples=10000,n_features=12,centers=10)" in order for the function call to complete correctly. The output in the example is also not easy to parse for a nonexpert. There is no output visualization, nor is there any apparent test quantifying goodness of fit (or some other metric) for how well the algorithm is working. I'm having trouble debugging this fully, but it looks like the visualization fails because at some point HAL calls for a plot using javascript, but this is run through a Python 2.7 call instead of Python 3 and the program fails to find the http.server module. I encountered these errors on a Mac with up to date versions of Python and all associated libraries.

Thank you for these suggestions.We made sure that HAL-x can be installed with the latest version of Python (3.9+). In the installation instructions we also clarified the installation for all packages:

```
source activate YOUR_CONDA_ENVIRONMENT

conda config --add channels conda-forge

conda install cython numpy fftw scipy

pip install hal-x
```

We also corrected the make_blobs syntax in the provided example. We thank the referee for pointing this out.

Finally we tested that the optional javascript visualization (as displayed in Figure S3) works on the latest MacOS. The http.server module is standard 3.6+ conda. In response to another referee, we also updated the source code to allow for using UMAP embedding instead of t-SNE (via the optional parameter embed_method='umap').

2. One of the main points of emphasis in the paper is the ability to scale the sensitivity of the analysis to obtain more coarse or granular clusters (point 3 on page 3). The authors present real and simulated examples demonstrating this principle, but it would be helpful if some more detail was included, especially for the real data example. The simulated example presented in Figure 3 (note: no information is provided about the source of the data) gives a sense for how this works, but it appears that the definition of clusters can be quite sensitive to the chosen accuracy value. A small shift in the cv-score cutoff from 0.97 to 0.98 causes two large clusters to merge together. Of course, part of this may be due simply to the nature of thresholds, but this is not easy to see from the example. It would be helpful for future users of this method if an example were provided where a wide range of values for the accuracy cutoff is explored in a smooth way. The real data example provides something like this (Figure 5C), but it is difficult to appreciate how clustering changes as the threshold shifts given the large steps in cv-score values. It would also be helpful if the authors could present more detail on their results in the main text. At present, in this example, little data is presented beyond broad, qualitative statements (e.g., "several of the key cell populations were only identified when the cv-score was 0.7 or lower"; which populations are these, and how is it inferred that these populations are important?). Practically, it would also be helpful if such a worked example was provided together with the code, so that users of the algorithm will be well-prepared to perform this analysis themselves.

We expanded the discussion on the relationship between the cv-score and clustering accuracy (F-score). In particular we re-arranged the 'proof-of-concept' figure into 6 panels (Figure 2), one for each synthetic dataset, in order to emphasize the monotonic relationship between our cv_score and F-score. In our modified figure 2, we added multiple small subset plots, to showcase how the clustering changes as the cv-score increases and the large number of generated clustering labels interpolate from an overclustering regime towards the ground-truth labels.

More broadly, we agree with referee #2 that clustering can be quite sensitive to cv-score, but we argue that this is highly dependent on the clustering task at hand. Some researchers might be more interested in finer clusterings. While we leave it to the user to select the cv-score that best suits their use case, we always provide the hierarchical relationship between the merged clusters to allow for further assessment. In fact, the driving philosophy behind our method is the idea that there is no single CV-score/F-score that works in all cases. Instead, this cut-off should be driven by biological intuition and/or significance, as defined by the experimentalist/user. By providing different potential clusters at different CV scores, the biologist is free to choose the most meaningful clustering for the question and dataset. Importantly, this also allows the experimentalist/user to explicitly check how this choice of CV score modifies their biological conclusions – hopefully allowing them to identify biologically robust phenomena.

3. In the section on tests on synthetic data, the authors mention the size of the data set that they consider, but nothing is stated about how the data is structured. This is in principle an important choice, especially given that this section gives a comparison between different methods on the data. The authors should describe this in detail, and ideally the test data should also be available online (either as part of the repository for the code, in a database such as Zenodo, or as part of the supplementary material for this paper).

Thank you for this suggestion. We are making our Lupus dataset in the Zenodo database:

10.5281/zenodo.6332934.

The synthetic dataset was generated using the ‘make_blobs’ function in Pythons’s scikit_learn library: these are points distributed in a multi-Gaussian distribution.

4. On page 7, the authors state that “we can also preprocess the input using an encoder if necessary” in place of t-SNE. At present, this statement is very vague, and it is not clear when the authors use t-SNE and when they use an encoder. How would the authors suggest that users of their software decide which approach to take for a particular data set? Can the consequences of different choices be explored on an example data set? How does this choice affect the run time?

We initially devised the encoder discussion especially to generalize the approach to clustering higher dimensional objects such as images. Since the objective of this paper is to focus on the analysis of single-cell biological samples, we removed the discussion/mentions of encoders throughout the manuscript. Please see the response to Comment 2 by Referee 1 who had similar concerns.

5. Similarly, the authors use multiple classifiers for different data sets. In the examples in Figure 1, the authors use support vector machines, while for the Lupus data set they use random forests. Why? On page 4, the authors state that “any reasonably powerful supervised learning algorithm” can be used, but again this statement is rather vague and leaves users to wonder whether different algorithms may be appropriate for different circumstances, or whether these approaches are truly interchangeable.

We apologize for this confusion. These choices reflect historical choices and not anything fundamental to the algorithm. For consistency, we have re-run figure 2 using random forest classifiers using n=100 estimators. For maximal flexibility, the source code lets the user select their appropriate classifier (choice between Naive Bayes, SVM, Random Forest, etc.). However, as shown in Figure 1, in order to obtain the decision boundaries shown in black, it requires at least a non-linear classifier (random forest was used in this case displayed in our new Figure 1). The statement made in the text refers to the idea that our method is robust as long as the classifiers used can fit the structure of the data provided. In our use cases, we found that using random forests appeared sufficiently powerful/complex for the data at hand. However, we could imagine that neural networks (CNN) might be better suited if one deals with images for instance. This is however beyond the scope of this work and is left for future work.

6. The authors claim that the accuracy of predicting clustering for out of sample data is a good proxy for what constitutes good clustering, and I agree with their intuitive rationale. However, this is a bit difficult to see in Figure 1 because there are quite a few clusterings with high out of sample accuracy and low F-scores. It appears that this might be due to plotting multiple different types of data sets on the same plot, but this is still somewhat difficult to see. How well-correlated are the out of sample accuracy and F-score for each type of data? Also, in the materials that I have available, the data sets themselves are not described. It would be very helpful if this information was included in the supplementary information. In particular, I am somewhat confused by the “no_structure” data set – how does one evaluate clustering accuracy on a data set that has no intrinsic structure?

We have thoroughly expanded the discussion and associated figures in order to clarify the monotonic relationship between traditional measures of clustering goodness such as F1-score and out-of-sample CV-score (discussed in reply to Comment 3 by Referee 1). With regards to the datasets used, we have added their description in the supplemental section. We also refer the referee to the benchmark page for clustering

methods for scikit-learn which inspired the use of these datasets: https://scikit-learn.org/stable/modules/clustering.html.

When plotting out-of-sample accuracy vs F-score on lin-log scale, we find a clear linear relationship which suggests high correlation. However, we want to emphasize that we are mostly interested in the monotonic relation for the scope of this work, i.e. higher cv score implies higher F-score with high probability. Finally, we have added a full description of the benchmark datasets in the supplemental material section.

7. Despite its frequent use as a metric of clustering quality in the paper, the (weighted) F1-score is never explicitly defined.

We have added a section in the supplemental material detailing the F1-score metric and referred to it as required throughout the manuscript in order to avoid confusion for the reader.

Minor comments:

1. On page 4, the authors write: "…there exist[s] specialized methods developed specifically for high-dimensional biological data such as Phenograph, others, etc." Here, Phenograph is the only method that is named. Are there other methods that should be listed here?

We thank the reviewer for this comment. We have added Phenograph, PARC, and FLowSOM to this list and referred readers to a paper comparing other early methods.

2. In Figures 4 and 5 many of the fonts are small and difficult to read. The clustering plots in Figure 5B are so small that it is very difficult to examine them in detail.

We have reworked these figures to make it easier to understand. Please see new Figure 6.

3. Given that the HAL-x software can produce visualizations, it would be helpful for users if some of these visualizations were included in plots in the paper. This requirement may already be satisfied – as explained above, I was not able to get plots to work with the software, and so I was not able to examine the output directly.

We have added a small subsection in the supplemental materials showcasing the javascript dashboard that can be generated via HAL. However, this is an experimental and completely optional feature that does not affect the output of HAL. As such. we prefer not to mention it directly in the main text.

4. It seems that equation 2 should be described as the negative log-likelihood (or minus the log-likelihood) instead of simply the log-likelihood.

Thank you for pointing this out. We have changed it to 'minus log-likelihood'.

Reviewer #3: In this paper, the authors proposed HAL-x, a new hierarchical density clustering algorithm that can be scalable to a large data. HAL-x is especially designed for flow cytometry data, a large single cell data that often suffers from the scalability problem. While HAL-x has multiple interesting components, there are still multiple components that are not sufficiently clarified. I provide my comments in detail below.

1. The degree of down-sampling seems to be data-dependent. However, this is not a trivial task. For example,

in the synthetic data analysis, the authors use very different values for different datasets, e.g., 10% vs. 0.5%. Can the authors provide some guideline to determine the degree of down-sampling?

Reviewer #3 is correct to point out that "the degree of down-sampling seems to be data-dependent". We present here a practical answer based on our use of HAL-X. Depending on the quality of the feature space (e.g. signal/noise of the antibody staining for CyTOF mass cytometric measurements), one can define few or a lot of clusters. In our experience, training the clustering models with as many cells as practical (typically, 200,000 cells in a 40-dimensional space of epitopes) is necessary to capture as much clustering diversity as possible. Higher number of cells can be extremely slow, lower number of cells might miss critical clusters of interest. In general, we have found that using a downsample training set of the order a 100,000 cells empirically balances the competing needs of not missing rare clusters while still maintaining computational tractability.

2. fitSNE is proposed to be used for the dimension reduction purpose. Recently UMAP became more popular compared to t-SNE-based approaches. Can the authors provide justification to use fitSNE rather than UMAP?

We primarily used FFT accelerated t-SNE because it provided speed of execution and reasonably good embeddings. Our agglomerative method is robust to changes in the low-dimensional embedding technique as long as regions of high density in the generated embedding represent true clusters in the high dimensional space. In order to study the effect of different embeddings, we modified the source code to allow for the use of UMAP [`McInnes et al, The Journal of Open Source Software, 2018`]. We added the option embed_method='tsne' or embed_method='umap'. We reran the code and obtained F-scores similar to the ones obtained via t-SNE. We also note that we found a 2x computational speed-up when using UMAP. We thank the referee for this suggestion.

3. It is proposed to map the data onto a two-dimensional plane using fitSNE. While 2D is great for the visualization purpose, it might not be optimal for the clustering purpose. Can the authors provide justification of using 2D or consider to use other dimensions?

The key reason for using low dimensional embeddings is because density estimation becomes accurate. We agree that 2D embeddings are particularly useful because they also allow for rapid visual validation. However, our method should work as long as density estimates can be performed. The fact that our algorithm yields similar clusters using both UMAP and tSNE suggests that the choice of embedding is not crucial. The underlying reason for this is that the algorithm starts by ``over clustering'' and then merging clusters using out-of-sample classification scores in the original high-dimensional space. However, as with many things in unsupervised learning, we are unable to provide a deeper theoretical justification for this beyond this intuition and empirical check.

4. In Equation 1, what is the mathematical meaning of arrow (->)? Convergence?

This arrow meant "in the limit where the number of data points approaches infinity". We have removed this ambiguous notation from the equation and we thank the referee for pointing this out.

5. In the kernel density estimation, kernel and bandwidth are two critical choices and they have significant impact on the output. In the case of kernel, while Gaussian kernel is popular, Epanechnikov kernel is more popular and mathematically considered to be optimal. Similarly, there are various bandwidth selectors,

including plug-in methods, cross-validation methods, etc. For example, Sheather & Jones method is a popular choice. Can the authors provide justification of using Gaussian kernel and a cross-validated MLE?

In our benchmark testing, we found that cross-validation MLE performed very well and was very fast. The benchmarking was done for the FDC (fast density clustering) package that was developed by one of the authors and that is available here : https://github.com/alexandreday/fast_density_clustering. FDC leaves it optional to the user to change kernel or fix their own bandwidth. We found that using Gaussian kernel + cross-validate MLE outperformed all out-of-the-box clustering methods from scikit learn and this the main reason we opted for these hyper-parameters (see main page of FDC github page linked above for comparisons).

6. In Prediction step, the cv-score cutoff is a critical tuning parameter, which determines the number of clusters. While I acknowledge that hierarchical clustering is the power of HAL-x, biologists often need to determine a specific number of clusters for downstream analyses. Can the authors provide any guideline to determine the number of clusters? Is the default value (0.5) recommended for the general purpose?

There is not a simple answer to this question. The number of clusters needs to be selected according to the biological problem at stake. On one hand, one wants the smallest number of clusters in order to increase the reliability of their definition (hence a high cv_score) but this brings the drawback of being potentially too coarse to capture the biological complexity of the system under consideration; on the other hand, lowering the cv_score hence increasing the number of clusters may better capture the variability of samples (e.g. by identifying a specific cluster whose frequency and/or phenotype best discriminates between two biological settings), but at the expense of reliability in the definition of clusters.

This is in fact a key rationale for our algorithm: one cannot decide a priori how many clusters one must define in the data in order to derive some biological insight. The need to rapidly cluster at different depths is the very feature being addressed with HAL-X. (see also reply to Comment 2 of Referee 2)

Note that the default value of cv_score=0.5 is in fact not recommended: it implies that the cross-validation of the cluster identification fails as a score of 0.5 practically implies random assignment during the training of the cluster classification.

7. For flow cytometry data, FlowSOM is the most popular algorithm for cell clustering and multivariate t-mixture is also likely popular. However, these popular algorithms are not included in the benchmarking. Please consider to include them in the benchmarking.

We have included FlowSOM in the benchmarking (see Figure 5).

8. I cannot find the Author Summary section. Please provide this section in the manuscript.

We apologize for this oversight. We have updated the author to include an author summary.
